# Comparison of Levetiracetam Versus Sodium Valproate as a Monotherapy in Early Childhood Epilepsy

**DOI:** 10.7759/cureus.87574

**Published:** 2025-07-09

**Authors:** Zainab Latif, Memoona Mukhtar, Ali Rohan, Waseem Farooq Shah, Zohaib Aslam, Maryam Akbar, Hafiz Muhammad Zeeshan Raza

**Affiliations:** 1 Pediatrics, Allama Iqbal Memorial Teaching Hospital, Sialkot, PAK; 2 Pediatrics, Gujranwala Medical College/Teaching Hospital, Gujranwala, PAK; 3 Internal Medicine, University College of Medicine and Dentistry, Lahore, PAK; 4 MD Pediatric Medicine, Registrar Pediatrics Department, Midcity Hospital, Lahore, PAK; 5 Pediatrics, University College of Medicine & Dentistry, University of Lahore, Lahore, PAK; 6 Pediatrics, Consultant Pediatric Medicine, Mayo Hospital Lahore, Lahore, PAK; 7 Bioinformatics, Commission on Science and Technology for Sustainable Development in the South (COMSATS) University Islamabad, Sahiwal, PAK

**Keywords:** antiepileptic drugs, childhood epilepsy, levetiracetam, seizure control, sodium valproate

## Abstract

Background: Childhood epilepsy requires long-term management with antiepileptic drugs (AEDs) that are both effective and well-tolerated. Although levetiracetam and sodium valproate are widely used as monotherapies, comparative data on their efficacy and safety in pediatric populations are limited. This study aimed to evaluate and compare their effectiveness and tolerability in children with epilepsy over six months.

Methods: A randomized controlled study was conducted involving 200 children aged 3 to 12 years with generalized or focal epilepsy. They were randomly assigned to receive either levetiracetam (n=100) or sodium valproate (n=100), and received sodium valproate at an initial dose of 30 mg/kg/day. Efficacy was defined as a threefold increase in seizure-free interval. Side effects were monitored monthly. Data were analyzed using SPSS version 29 with a significance threshold of p<0.05.

Results: Seizure control was achieved in 85% of children in the levetiracetam group and 73% in the sodium valproate group (p=0.037). Absence of side effects was reported in 31% of the levetiracetam group and 10% of the sodium valproate group (p=0.012). Weight gain occurred in 16 children (16.0%) in the sodium valproate group versus 5 children (5.0%) in the levetiracetam group. Other side effects, including hepatotoxicity, rash, somnolence, tremors, and behavioral changes, were comparable between the groups.

Conclusion: Levetiracetam appears to be more effective and better tolerated than sodium valproate for the management of pediatric epilepsy. These findings suggest that levetiracetam could be considered a preferred first-line monotherapy. However, further longitudinal studies are warranted to validate these outcomes over longer treatment durations.

## Introduction

Epilepsy is one of the most prevalent chronic neurological disorders, affecting an estimated 50 million individuals worldwide, across all ages, geographic regions, and socioeconomic statuses [1]. The point prevalence is estimated to range between 4 and 10 per 1,000 persons, making epilepsy a major public health concern globally [2]. The incidence is approximately 50 to 60 new cases per 100,000 person-years, and the lifetime risk is estimated to be up to 8% of individuals will experience at least one epileptic seizure [3]. These figures underscore the substantial burden epilepsy imposes on individuals, families, and healthcare systems, particularly in low-resource settings [3].

In children, epilepsy represents a major contributor to neurological morbidity [4]. It is defined by a persistent tendency to produce epileptic seizures, which arise from abnormal and synchronized neuronal activity within the brain [5]. Seizures may manifest with observable convulsions, altered awareness, or motor disturbances. Alternatively, they may occur as electrographic seizures detectable only via electroencephalography (EEG) [6]. Seizure types are typically categorized into generalized and focal (partial) types, each necessitating specific pharmacological approaches depending on their clinical and electrographic features [7].

The mainstay of epilepsy management is pharmacological therapy with antiepileptic drugs (AEDs) selection based on seizure type, patient age, comorbidities, side effects, and drug interactions [8]. Widely used AEDs include sodium valproate, carbamazepine, phenytoin, and lamotrigine [8]. Although many of these agents offer comparable efficacy, drug selection in pediatric epilepsy must carefully balance efficacy with safety, tolerability, cognitive side-effect profiles, and long-term developmental considerations [9].

Sodium valproate has traditionally served as a broad-spectrum agent effective against both generalized and focal seizures [10]. However, its use has raised concerns due to notable adverse effects such as hepatotoxicity, weight gain, tremors, and teratogenicity, particularly relevant in female patients of childbearing potential [10]. Over the past decade, levetiracetam has emerged as a promising alternative due to its favorable pharmacokinetic profile, minimal drug interactions, and tolerability [11]. Levetiracetam is increasingly used off-label as monotherapy in children, including those with treatment-resistant epilepsy, and growing evidence supports its efficacy across a broad spectrum of seizure types [11].

Nevertheless, head-to-head comparative data between levetiracetam and traditional agents like sodium valproate remain limited, especially when used as initial monotherapy in newly diagnosed pediatric cases [12]. This gap is particularly relevant in resource-limited settings, where cost-effectiveness and safety play a pivotal role in clinical decision-making. To address this need, the present randomized controlled trial was designed to compare the efficacy and safety of levetiracetam versus sodium valproate in controlling seizures in children with generalized or focal epilepsy, with the goal of informing evidence-based treatment decisions in pediatric neurology.

## Materials and methods

Study design and setting

This randomized controlled study was conducted from September 2024 to March 2025 to assess the efficacy and safety of levetiracetam and sodium valproate as monotherapy in children with epilepsy. The study was carried out at the Pediatric Epilepsy Clinic of Allama Iqbal Memorial Teaching Hospital and Government Sardar Begum Teaching Hospital, affiliated with Khawaja Muhammad Safdar Medical College, Sialkot, Pakistan.

Participants

Children aged 3 to 12 years, of either sex, diagnosed with generalized or focal epilepsy or epileptic syndromes, were eligible for inclusion. Participants were recruited regardless of socioeconomic status or growth parameters. Inclusion criteria required either a new diagnosis of epilepsy or no use of AEDs for at least three months prior to enrollment. Exclusion criteria included congenital neurological anomalies, a history of recent head trauma, prior use of AEDs for more than 15 days, known liver disease, or refusal to provide informed consent.

Randomization and intervention

The sample size was calculated using the WHO sample size calculator for comparing two proportions in a randomized controlled trial. The calculation was based on an assumed efficacy rate of 85% for levetiracetam and 70% for sodium valproate, derived from previously published pediatric epilepsy studies. To detect this 15% absolute difference in seizure control rates, with a power of 80% (β = 0.20) and a significance level of 5% (α = 0.05, two-sided), the required sample size was calculated as 94 participants per group. To account for potential loss to follow-up or incomplete data (estimated at 5-6%), the final sample size was rounded up to 100 children per group. This resulted in 200 children, randomly assigned to two equal groups. Group A (n = 100) received levetiracetam, and Group B (n = 100) received sodium valproate. Randomization was performed only after eligibility was confirmed and informed consent obtained. Both drugs were given orally, as tablets or suspension, at an initial dose of 30 mg/kg/day. A seizure management booklet containing guidance for caregivers on handling acute seizure episodes was distributed to all families.

Follow-up and dose adjustment

Participants were followed monthly for six months. In cases of breakthrough seizures, the antiepileptic dose was titrated upward in increments of 10 mg/kg/day. Compliance was monitored through direct caregiver interviews and verification of medication bottles at each follow-up visit.

Baseline and clinical assessments

At baseline, each child underwent a detailed physical examination and completed a structured clinical questionnaire. Baseline investigations included standard laboratory tests and EEG, where feasible. Seizure characteristics, including frequency, type, and duration, were recorded and monitored using seizure diaries maintained by caregivers throughout the study duration.

Outcome measures

The primary outcome was seizure control, defined as a threefold increase in the seizure-free interval compared to baseline. Secondary outcomes included the occurrence and profile of adverse drug reactions. Efficacy was evaluated based on the proportion of participants who achieved seizure freedom, while tolerability was assessed through the frequency and severity of reported adverse effects.

Statistical analysis

Data were analyzed using SPSS version 29. Descriptive statistics were used to summarize demographic and clinical characteristics. Frequency distributions were examined to clean the data and identify outliers. Group comparisons for seizure control and adverse effects were conducted using the chi-square test. A p-value of less than 0.05 was considered statistically significant.

Ethical approval

The study protocol was reviewed and approved by the Institutional Review Board of Khawaja Muhammad Safdar Medical College, Sialkot (Approval number: 16/RCE/KMSMC). Written informed consent was obtained from the guardians of all participants prior to enrollment in the study.

## Results

Demographic and diagnostic outcomes

The gender distribution was similar between groups: males comprised 54% of Group A and 52% of Group B; females accounted for 46% and 48%, respectively. The mean age was 7.38 ± 2.82 years in Group A and 7.84 ± 2.82 years in Group B. Mean age at seizure onset was 7.24 ± 3.45 years and 6.85 ± 3.76 years, respectively. Socioeconomic status was comparable, with low-income patients at 40 (40.0%) in Group A and 41 (41.0%) in Group B, middle-class patients at 32 (32.0%) in both groups, and high-income patients at 28 (28.0%) in Group A and 27 (27.0%) in Group B. Family history of epilepsy was noted in 26 (26.0%) of Group A and 25 (25.0%) of Group B, while 74 (74.0%) and 75 (75.0%) had no family history. Focal seizures were observed in 56 (56.0%) of Group A and 54 (54.0%) of Group B, whereas generalized seizures were recorded in 44 (44.0%) and 46 (46.0%), respectively. Regarding associated features, 21 (21.0%) in Group A and 23 (23.0%) in Group B had no associated symptoms, aura was noted in 19 (19.0%) and 17 (17.0%), frothing in 21 (21.0%) and 19 (19.0%), post-ictal dizziness in 20 (20.0%) and 18 (18.0%), and tongue bite in 19 (19.0%) and 23 (23.0%), respectively (Table 1).

**Table 1:** Comparison of the distribution of different variables between groups

Variables		Groups	
		Group A (Levetiracetam)	Group B (Sodium valproate)
Gender	Male	54 (54.0%)	52 (52.0%)
	Female	46 (46.0%)	48 (48.0%)
Age	Age (years)	7.38±2.82	7.84±2.82
	Age at onset	7.24±3.45	6.85±3.76
Socio-economic status	Low	40 (40.0%)	41 (41.0%)
	Middle	32 (32.0%)	32 (32.0%)
	High	28 (28.0%)	27 (27.0%)
Family history	Yes	26 (26.0%)	25 (25.0%)
	No	74 (74.0%)	75 (75.0%)
Type of seizure	Focal	56 (56.0%)	54 (54.0%)
	Generalized	44 (44.0%)	46 (46.0%)
Associated features	None	2 1(21.0%)	23 (23.0%)
	Aura	19 (19.0%)	17 (17.0%)
	Frothing	21 (21.0%)	19 (19.0%)
	Post-ictal dizziness	20 (20.0%)	18 (18.0%)
	Tongue bite	19 (19.0%)	23 (23.0%)

Comparative outcomes of efficacy

In Group A, 85 (85.0%) of patients remained seizure-free, while 15 (15.0%) experienced repeat seizures. In Group B, 73 (73.0%) achieved seizure control, whereas 27 (27.0%) had recurrent seizures. The p-value of 0.037 suggests a statistically significant difference, indicating that levetiracetam was more effective in seizure control compared to sodium valproate (Table 2).

**Table 2:** Comparison of efficacy between both groups Comparison of treatment efficacy between groups using the Chi-square test (χ² = 4.34, p = 0.037). The chi-square value (4.34) was computed based on a 2x2 contingency table of seizure control rates.

Efficacy	Group A (Levetiracetam)	Group B (Sodium Valproate)	Chi-square	p-value
Seizure-free	85 (85.0%)	73 (73.0%)	4.34	0.037
Not seizure-free	15 (15.0%)	27 (27.0%)		
Total	100 (100.0%)	100 (100.0%)		

Comparative outcomes of side effects

No side effects were reported in 31 (31.0%) of Group A compared to only 10 (10.0%) of Group B, with a statistically significant p-value of 0.012. Other side effects were generally comparable between the groups. In Group A vs. Group B, the frequencies were altered behavior: 11% vs. 14%, anorexia: 9% vs. 9%, hepatotoxicity: 9% vs. 12%, rash: 13% vs. 12%, somnolence: 13% vs. 17%, tremors: 9% vs. 10%. Weight gain was significantly more common in Group B at 16 (16.0%) compared to 5 (5.0%) in Group A (Table 3). These findings suggest that levetiracetam had a more favorable side-effect profile, with significantly more patients experiencing no side effects and fewer instances of weight gain compared to sodium valproate.

**Table 3:** Comparison of side effects between both groups

Side Effect	Group A(Levetiracetam)	Group B(Sodium Valproate)	Chi-square / Fisher’s exact	p-value
None	31 (31.0%)	10 (10.0%)	χ² = 10.24	0.0014
Altered behavior	11 (11.0%)	14 (14.0%)	χ² = 0.38	0.5376
Anorexia	9 (9.0%)	9 (9.0%)	χ² = 0.00	1.0000
Hepatotoxicity	9 (9.0%)	12 (12.0%)	χ² = 0.46	0.4961
Rash	13 (13.0%)	12 (12.0%)	χ² = 0.05	0.8216
Somnolence	13 (13.0%)	17 (17.0%)	χ² = 0.68	0.4084
Tremors	9 (9.0%)	10 (10.0%)	χ² = 0.06	0.8062
Weight gain	5 (5.0%)	16 (16.0%)	χ² = 6.53	0.0106
Total	100 (100.0%)	100 (100.0%)		

## Discussion

This study found that levetiracetam is more effective in controlling seizures in children with epilepsy than the other antiepileptic drug examined (sodium valproate). The rate of efficacy in the levetiracetam group was significantly (p=0.037) higher (85%) than in the sodium valproate group (73%). Previous studies that showed levetiracetam as a monotherapy for pediatric epilepsy are in agreement with the results of this study [13].

Levetiracetam’s superior seizure control can be attributed to its unique mechanism of action, which involves binding to synaptic vesicle protein 2A, thereby modulating neurotransmitter release [13]. In contrast, sodium valproate exerts its antiepileptic effects through multiple mechanisms, including enhancement of gamma-aminobutyric acid (GABA) transmission and inhibition of voltage-gated sodium channels [14]. However, despite its broad-spectrum activity, valproate has been associated with higher rates of breakthrough seizures and adverse effects, particularly in long-term use [15].

In terms of safety, 31% of patients in the levetiracetam group reported no side effects, compared to only 10% in the sodium valproate group (p=0.012). Notably, weight gain was significantly more common with sodium valproate (16% vs. 5%), a finding consistent with prior research highlighting valproate-induced metabolic changes and increased appetite. This weight gain is particularly concerning in pediatric patients due to its potential impact on long-term health, including the risk of obesity and metabolic syndrome [16-17].

Other adverse effects, including somnolence, hepatotoxicity, rash, and tremors, were comparable between the two groups. However, behavioral disturbances such as irritability and aggression were reported more frequently with levetiracetam, as previously documented in the literature [18]. Nonetheless, these side effects were generally mild and manageable, and none of the patients required treatment discontinuation. The results of this study support the use of levetiracetam as a first-line monotherapy for pediatric epilepsy due to its higher efficacy and better tolerability profile. These findings are in agreement with large-scale trials that have demonstrated levetiracetam's comparable or superior seizure control and lower risk of adverse effects relative to traditional AEDs, including sodium valproate [19-20]. However, some studies have suggested that levetiracetam may be less effective in specific epilepsy syndromes, such as generalized absence epilepsy, where valproate remains a preferred choice [21].

One limitation of this study is the relatively short follow-up period of six months, which may not capture the long-term effects of either drug. Additionally, EEG findings were not consistently available for all patients, limiting the ability to correlate treatment response with specific electrophysiological patterns. Future research should include longer follow-up periods and multi-center trials to validate these findings and assess the impact of AEDs on cognitive and behavioral outcomes in children.

## Conclusions

Levetiracetam demonstrated superior efficacy in seizure control and a more favorable safety profile compared to sodium valproate in the pediatric population. These findings support levetiracetam as a potentially preferable first-line monotherapy for children with generalized or focal epilepsy, particularly due to its lower incidence of adverse effects and better overall tolerability. However, further large-scale and long-term studies are warranted to confirm these results and to evaluate the sustained efficacy, neurodevelopmental outcomes, and safety of levetiracetam over extended treatment durations.
